# *In silico* Exploration of Interactions Between Potential COVID-19 Antiviral Treatments and the Pore of the hERG Potassium Channel—A Drug Antitarget

**DOI:** 10.3389/fcvm.2021.645172

**Published:** 2021-05-04

**Authors:** Ehab Al-Moubarak, Mohsen Sharifi, Jules C. Hancox

**Affiliations:** ^1^School of Physiology, Pharmacology and Neuroscience, University of Bristol, Biomedical Sciences Building, University Walk, Bristol, United Kingdom; ^2^Independent Scientist, Indianapolis, IN, United States

**Keywords:** hERG, human ether-à-go-go–related gene, antiviral, atazanavir, lopinavir-ritonavir, remdesivir, favipiravir

## Abstract

**Background:** In the absence of SARS-CoV-2 specific antiviral treatments, various repurposed pharmaceutical approaches are under investigation for the treatment of COVID-19. Antiviral drugs considered for this condition include atazanavir, remdesivir, lopinavir-ritonavir, and favipiravir. Whilst the combination of lopinavir and ritonavir has been previously linked to prolongation of the QT_c_ interval on the ECG and risk of *torsades de pointes* arrhythmia, less is known in this regard about atazanavir, remdesivir, and favipiravir. Unwanted abnormalities of drug-induced QT_c_ prolongation by diverse drugs are commonly mediated by a single cardiac anti-target, the hERG potassium channel. This computational modeling study was undertaken in order to explore the ability of these five drugs to interact with known determinants of drug binding to the hERG channel pore.

**Methods:** Atazanavir, remdesivir, ritonavir, lopinavir and favipiravir were docked to *in silico* models of the pore domain of hERG, derived from cryo-EM structures of hERG and the closely related EAG channel.

**Results:** Atazanavir was readily accommodated in the open hERG channel pore in proximity to the S6 Y652 and F656 residues, consistent with published experimental data implicating these aromatic residues in atazanavir binding to the channel. Lopinavir, ritonavir, and remdesivir were also accommodated in the open channel, making contacts in a model-dependent fashion with S6 aromatic residues and with residues at the base of the selectivity filter/pore helix. The ability of remdesivir (at 30 μM) to inhibit the channel was confirmed using patch-clamp recording. None of these four drugs could be accommodated in the closed channel structure. Favipiravir, a much smaller molecule, was able to fit within the closed channel and could adopt multiple binding poses in the open channel, but with few simultaneous interactions with key binding residues. Only favipiravir and remdesivir showed the potential to interact with lateral pockets below the selectivity filter of the channel.

**Conclusions:** All the antiviral drugs studied here can, in principle, interact with components of the hERG potassium channel canonical binding site, but are likely to differ in their ability to access lateral binding pockets. Favipiravir's small size and relatively paucity of simultaneous interactions may confer reduced hERG liability compared to the other drugs. Experimental structure-function studies are now warranted to validate these observations.

## Introduction

The coronavirus disease of 2019 (COVID-19), caused by the SARS-CoV-2 virus, poses an unprecedented challenge to modern healthcare systems. Although vaccines are now emerging [e.g., (1–4)], logistical challenges in production and global administration of billions of vaccine doses and the potential for incomplete vaccine take-up and efficacy mean that therapeutic treatments are also needed. Since the emergence of SARS-CoV-2, considerable effort has been invested to identify existing drugs that may be successfully repurposed for treatment of the illness. The antimalarial agents chloroquine and hydroxychloroquine were initially reported to be effective against SARS-CoV-2 *in vitro* (5, 6). However, whilst some studies have reported potential clinical benefit of these drugs [e.g., (7–9)], others are inconsistent with benefit [e.g., (10–12)] and there is a risk of QT interval prolongation and ventricular arrhythmia, particularly at higher concentrations (12–14). Other potential Covid-19 repurposed treatments include antivirals originally developed for other conditions (15, 16). The use of an *in silico* drug target deep-learning model has suggested a number of antiviral agents to be able to inhibit the 3C-like proteinase of SARS-CoV-2, including the antiretrovirals atazanavir and lopinavir and the broad spectrum antiviral agent remdesivir (17). Lopinavir is used in combination with ritonavir (which increases lopinavir half-life through inhibition of cytochrome P450) to treat human immunodeficiency virus and there is some evidence of efficacy against SARS-CoV-1 and MERS-CoV (15, 16). Initial randomized control trial data have not provided evidence for a benefit of the lopinavir-ritonavir combination beyond standard care, in patients hospitalized with severe COVID-19 (18). Remdesivir is a broad spectrum antiviral that has been found to be effective against diverse types of β coronaviruses (19). Intravenous remdesivir is undergoing clinical investigation in patients hospitalized with COVID-19 and initial trial data have shown a trend toward a reduction in time to clinical improvement (20, 21), warranting further study. Favipiravir is a broad spectrum antiviral agent shown to inhibit replication of a substantial number of RNA viruses (22). Favipiravir's efficacy against SARS-Cov-2 has been demonstrated pre-clinically in a Syrian Hamster model in which the drug reduced lung viral titers and alleviated disease (23). A recent open label study of its use in COVID-19 has reported an association between favipiravir and a shorter viral clearance time and, following adjustment for confounders, improved chest imaging (24). Whilst further study is needed, a recent scoping review has concluded that both remdesivir and favipiravir may be promising treatments for COVID-19 (25).

A proportion of COVID-19 patients have cardiac damage and concerns have been expressed regarding the risk of proarrhythmic effects of potential COVID-19 treatments, particularly in relation to producing prolongation of the rate corrected QT (QT_c_) interval and associated *torsades de pointes* (TdP) arrhythmia (26–28). Whilst the emerging clinical data clearly support such a risk for chloroquine/hydroxychloroquine (12–14), it may also occur for some antivirals. TdP requiring resuscitation has been reported for a critically ill COVID-19 patient treated with remdesivir (29). Lopinavir/ritonavir and atazanavir have previously been associated with QT prolongation and TdP in the absence of COVID-19 (30–32). Favipiravir has been reported not to affect the QT/QT_c_ interval in healthy adults (33), although mild QT_c_ interval prolongation has been reported in a patient infected by Ebola-virus (34). Nearly all drugs associated with QT_c_ interval prolongation and TdP inhibit the cardiac hERG (*human Ether-à-go-go Related Gene*) potassium channel, which mediates the rapid delayed rectifier K^+^ current, I_Kr_; I_Kr_ is a key determinant of ventricular repolarisation (35, 36). The association between TdP/QT_c_ interval prolongation and pharmacological inhibition of hERG channels is sufficiently strong that testing for pharmacological inhibition of hERG channels is a key component of safety testing of candidate pharmaceuticals (36, 37). The consequences of pharmacological blockade of hERG may be exacerbated in hyperinflammatory states, since interleukin-6 can inhibit I_Kr/_hERG via the Janus Kinase pathway (38) and the risk of arrhythmia may increase with severity of infection/inflammation (27). Lopinavir, ritonavir, and atazanvir have been reported to be able to inhibit hERG channel current (30, 39). However, at the time of writing, there are no peer reviewed studies of the ability of remdesivir or favipiravir to interact with the hERG channel. Whilst such information would be valuable, the SARS-CoV-2 pandemic has interfered with much laboratory-based experimental activity. The recent availability of a cryo-EM structure of the hERG channel (40) provides a means to investigate *in silico* the ability of drugs to interact with known molecular determinants of drug binding to the channel (41). Accordingly, this computational modeling study was undertaken to probe interactions between each of atazanavir, ritonavir, lopinavir, remdesivir, favipiravir, and constituents of the canonical drug binding site within the hERG channel pore. Our findings suggest that all of these agents can, in principle, interact with components of the hERG potassium channel canonical binding site, but with some drug-specific differences in the observed interactions.

## Materials and Methods

Docking simulations used the recent Cryo-EM structure of the open pore state of hERG channel (40), PDB: 5VA2 and two closely related open pore models. These models were developed to predict more favorable hERG pore conformations for drug binding using molecular dynamics (MD) simulations starting from the available Cryo-EM hERG structure with the aim of presenting important F656 side chains into a pore-facing conformation to interact with drugs. The F656 residue is well-known to be an important determinant of drug-hERG channel interactions and its position relative to pore varies between models (41). The published Dickson model was obtained from MD simulations in the presence of hERG inhibitors (42). The in-house model was obtained from a short MD simulation in which the F656 side chain of one of the four hERG subunits was found to reorient toward the pore—this subunit was then replicated around all four pore subunits to produce a model with all four F656 side chains facing the pore. Molecular dynamics simulation of the hERG membrane domain, which underpinned the in-house model, was conducted using the cryoEM structure of hERG (PDB:5VA2) with several extracellular loops of missing atom density modeled into the structure using Modeler 9.17 (43) and the N- and C-terminal cytoplasmic domains removed. Unrestrained MD of the hERG membrane domain model was run in a POPC bilayer patch (385 lipids: 127 × 133 angstroms) with water layers (150 mM NaCl) above and below the membrane resulting in a total depth of the periodic boundary system of 120 angstroms. MD simulations were carried out at 310 K as described in (44), using Gromacs 5.1.4 with the amber99sb-ildn force field for protein and the SLipids force field for POPC (45, 46). The use of two MD-based open hERG models along with the Cryo EM structure was anticipated to give the opportunity to explore antiviral binding in different conformations of the canonical binding site (and in particular the position of the F656 with respect to the pore). A series of docking simulations was also performed with a hERG closed pore model based on rat EAG closed pore cryo-EM structure as described previously (47). Antiviral structures were converted from SMILE representation (obtained from PubChem database) to 3D structures and then hydrogens added and energy minimized using Molecular Operating Environment (MOE).

Antiviral molecules were docked in each of the hERG structures and models using GOLD (version v2020.1; Cambridge Crystallographic Data Centre, Cambridge, UK). The central pore cavity was chosen as a binding site where a radius of 10 angstrom extended from the centre of the cavity and in a level with a middle point between the canonical aromatic residues F656 and Y652. The side chains of these aromatic residues were allowed to be freely flexible during docking simulations. The antiviral ligands were also fully flexible during the molecular docking studies. Rotamer sampling was maximally set to 300,000 generations. Docking was scored by Goldscore and rescored with ChemScore scoring function. Two-hundred docking runs were made in each case and the low-energy-score poses were retained and inspected. Antiviral molecules were also docked within a side pocket under the selectivity filter in the open pore F656-rotated hERG model. This binding pocket was centred above the β-carbon of Y652 and encompassed a volume having a radius of 7 angstroms. Amino acid side chains that comprise the putative canonical binding site and binding pocket were allowed to rotate freely during docking runs to accommodate the drug. Thus, the side chains for the following residues from chain A were allowed to rotate freely: F557, L622, T623, S624, L650, M651, Y652, I655. F656 from chains A and B were also allowed to rotate. Similar settings and parameters were used as above where also 200 docking repeats for each drug were generated and low energy poses were considered.

A further independent set of docking simulations was performed using MOE suite using the Cryo-EM structure and the two open pore models. Fifty docking repeats were performed for each potential antiviral compound in the central cavity binding site. The hERG channel structures were prepared and 3D-protonated followed by performing tethered energy minimization prior to commencement of docking. Docking regions were biased by selection of key residues in the canonical and lateral binding site (namely F656, Y652, T623, S624; and additionally, the following residues: F557, M651) with a further nine angstroms from selection. Energy-minimized (using an all-atom forcefield combining Amber12 and parameterized for small molecules using 2D Extended-Hückel-Theory method). Antiviral ligands were then docked in each of three hERG structures. The GBVI/WSA ΔG scoring function was used which is a forcefield-based scoring function that estimates the free energy of binding of the ligand from a given pose.

The results are visualized using PyMOL Molecular Graphics System, Version 2.0 Schrödinger, LLC.

Functional hERG channels are comprised of four identical protein subunits (designated here A, B, C, D; see Supplementary Figure 1). As some drug-channel interactions involved residues from different subunits in places the results text refers to the Chain ID when identifying amino acid and in such cases, the subunit ID is given before the residue ID, (e.g., C:F557). Details of the interactions are described in Supplementary Figure 1 (see online supplement).

Patch clamp experiments to investigate remdesivir inhibition of hERG ionic current (I_hERG_) were performed on HEK 293 cells stably expressing WT hERG. Remdesivir (purchased from Medkoo Biosciences) was dissolved in dimethyl sulfoxide (DMSO) to produce a stock solution of 30 mM and was applied at a 1/1,000 dilution (30 μM) in Tyrode's solution. Recordings were made at 37°C (whole cell patch clamp) using an Axopatch 200B amplifier (Molecular Devices) with a CV-4/100 headstage and data acquisition via a Digidata 1320 interface (Molecular Devices). The extracellular superfusate was a standard Tyrode's solution containing (in mM): 140 NaCl, 4 KCl, 2.5 CaCl_2_, 1 MgCl_2_, 10 glucose, and 5 HEPES (titrated to pH 7.4 with NaOH) (48–50). Patch pipettes (AM-systems Inc, USA) had resistances of 2–4 MΩ and were filled with a solution containing (in mM): 130 KCl, 1 MgCl_2_, 5 EGTA, 5 MgATP, and 10 HEPES (titrated to pH 7.2 with KOH) (48–50). Series resistance was typically compensated 60–80%. Currents were filtered at 2 kHz and were digitized at 10 kHz. Data are presented as mean ± SEM of the number of independent experiments indicated (*n*) after analysis.

## Results

### Atazanavir

**Figure 1:**
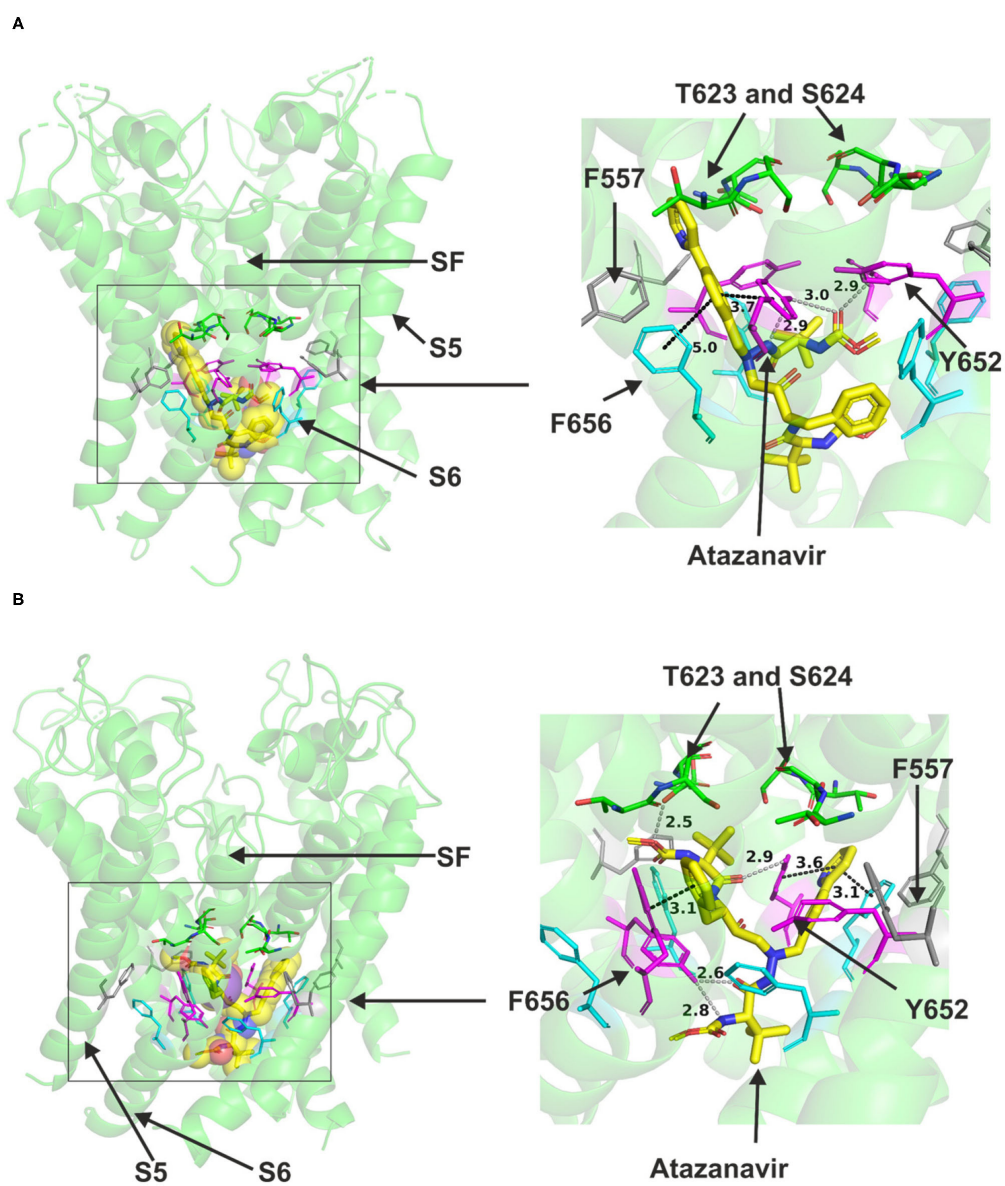
Location of the assigned binding site of atazanavir in the hERG cryo-EM structure and the closely related Dickson et al. model with the drug docked in the configuration shown in the methods section. The complete structure in **(A)** and **(B)** illustrates the location of segment 6 (S6) lining the pore where key residues such as Y652 and F656 are located and the outer S5 segment where F557, a major residue of the side pocket, is located. The selectivity filter region (SF) is also annotated where T623 and S624 sit at the base of the pore helix attached to it. Atazanavir is represented as a space-filling structure in which carbon atoms are represented in yellow, nitrogen in blue, oxygen in red. Major amino acid constituents of the binding sites were shown as sticks. In all structure figures, the hERG pore amino acid side chains are colored as follows: Phe-557, *gray*; Thr-623 and Ser-624, *green*; Tyr-652, *pink*; and Phe-656, *blue*. Atazanavir is shown in *yellow*. **(A,B)** show low-energy-score pose for atazanavir docked into the hERG pore with docking biased to promote occupation of canonical binding site. Annotations (dotted lines) define potential interactions between drug and amino acid side chains, distances in angstroms between the drug molecule and key residues were written adjacent to each dotted line. **(A)** shows the atazanavir docking in the hERG cryo-EM structure. **(B)**, shows the atazanavir docking in the Dickson et al. model based on hERG cryo-EM structure. This run in the Dickson et al. model is particularly important since rotamers of at least one of Phe-656 side chains was selected to orient the side chain Cα-Cβ bond toward the pore.

Atazanavir could readily be accommodated in the canonical central cavity binding site in hERG open pore structure (Figure 1); however, due to its size, it did not fit in hERG closed pore model in which the central cavity became significantly smaller compared to that in the open state. Docking the drug to both the cryo EM structure and the closely related MD-based model of hERG by Dickson et al. suggested that direct binding interactions occur between the molecule and the channel (Figure 1). In low energy poses, atazanavir was found in proximity to canonical aromatic residues F656 and Y652 in both models (Figure 1B) illustrates this for the Dickson et al. model. This is in good agreement with experimental observations for atazanavir (39). The drug also approached T623 and S624 near the base of the selectivity filter/pore helix. Atazanavir was also able to contact a serine residue (S660) one turn lower than F656 toward the cytoplasmic opening of the channel. Further details of predicted interactions are described in Supplementary Figure 2.

### Lopinavir and Ritonavir

**Figure 2:**
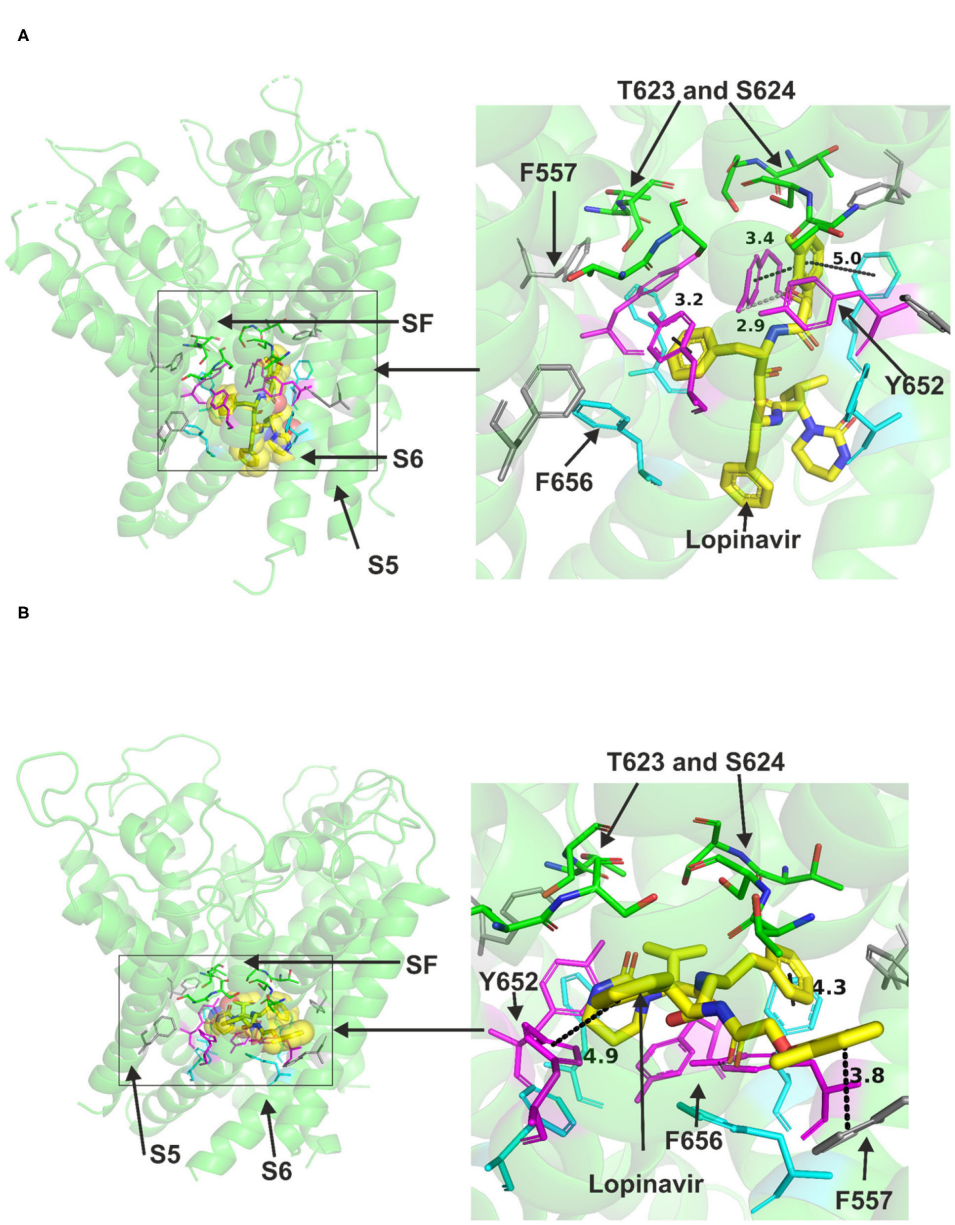
Location of the assigned pore binding site of lopinavir in the hERG cryo-EM structure and the closely related Dickson et al. model with the drug docked in the configuration shown in the methods section. The complete structure in **(A,B)** shows the location of segment 6 (S6) lining the pore where key residues such as Y652 and F656 are located and the outer S5 segment where F557, a major residue of the side pocket, is located. The selectivity SF was also annotated where T623 and S624 sit at the base of the pore helix attached to it. Lopinavir is represented as a space-filling structure. Major amino acid constituents of the binding sites were shown as sticks. Binding residues and atoms of the drug molecule colored as for Figure 1. **(A,B)** show low-energy-score pose for lopinavir docked into the hERG pore with docking biased to promote occupation of the canonical binding site. **(A)** shows the lopinavir docking in the hERG cryo-EM structure. **(B)** shows the lopinavir docking in the Dickson et al. model based on hERG cryo-EM structure. Annotations (dotted lines) define potential interactions between drug and amino acid side chains, distances in **(A)** between the drug molecule and key residues were written adjacent to each dotted line. This run in the Dickson et al. model is particularly important since rotamers of at least one of F656 side chains was selected to orient the side chain Cα-Cβ bond toward the pore.

Lopinavir could be accommodated in the central cavity of the hERG open pore structure and models (Figure 2). The drug interacted with the channel mainly via hydrophobic interactions. F656 and Y652 in S6 were able to interact with the drug in the low energy poses in the cryo-EM structure and in the open pore in-house model. However, docking the drug molecule to the open channel model by Dickson et al. showed the possibility that a part of the drug molecule extended toward the side pocket and interact with F557 in S5 (Figure 2B). Despite the ability of part of the drug molecule to stretch further to the side pocket, it was still able to contact key residues in the canonical binding site (Supplementary Figure 3). The docking also showed the possibility of the drug to form strong hydrogen bonds, mainly with S624, F656, and Y652 residues (Supplementary Figure 2).

**Figure 3:**
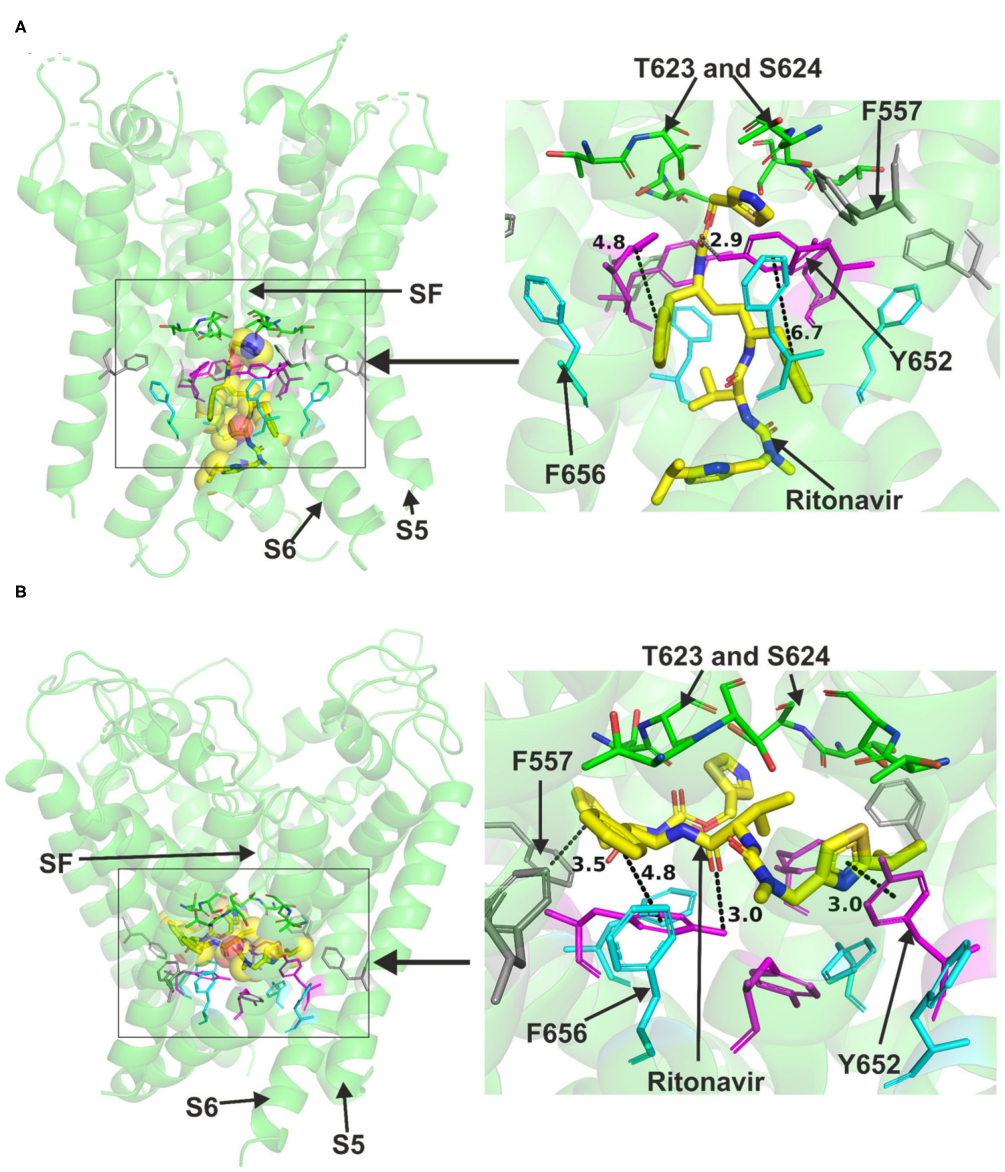
Location of the assigned pore binding site of ritonavir in the hERG cryo-EM structure and the closely related Dickson et al. model with the drug docked in the configuration shown in the methods section. The complete structure in **(A,B)** shows the location of segment 6 (S6) lining the pore where key residues such as Y652 and F656 are located and the outer S5 segment where F557, a major residue of the side pocket, is located. The selectivity SF was also annotated where T623 and S624 sit at the base of the pore helix attached to it. Ritonavir is represented as a space-filling *yellow* surface. Major amino acid constituents of the binding sites were shown as sticks in the box. Binding residues and atoms of drug molecule colored as for Figure 1. **(A,B)** show low-energy-score pose for ritonavir docked into the hERG pore with docking biased to promote occupation of canonical binding site. Annotations (dotted lines) define potential interactions between drug and amino acid side chains, distances in **(A)** between the drug molecule and key residues were written adjacent to each dotted line. **(A)** Shows the ritonavir docking in the hERG cryo-EM structure. *B* Shows the ritonavir docking in the Dickson et al. model based on hERG cryo-EM structure. This run in the Dickson et al. model is particularly important since rotamers of at least one of F656 side chains was selected to orient the side chain Cα-Cβ bond toward the pore.

Ritonavir was also readily accommodated in the canonical binding site of hERG when docked to the Cryo-EM structure or the Dickson et al. model, both representing the open pore state of the channel (Figure 3). Docking ritonavir to the cryo-EM structure resulted in association with several central cavity residues including Y652, S660 in S6 and S624 in the pore helix (details are presented in Supplementary Figure 4). Docking the drug in the Dickson et al. model revealed slightly different pose with a part of the drug molecule able to advance near the peripheral residue 557 in S5 like lopinavir. In this pose, Y652 was also able to interact with ritonavir. T623, S624 in the pore helix and F656 in S6 could also interact with the drug via hydrogen bonds. Both binding models indicated the ability of Y652 to interact with the sulfur atom in a thiazole group within the ritonavir molecule.

Neither lopinavir nor ritonavir could be docked to the closed pore model of hERG. Attempts were also made to dock each of the two drugs to a side pocket under the selectivity filter in the in-house open pore model but could not be accommodated. However, as introduced, small part of these structures could advance to this binding pocket while the majority parts of the molecules were still in the canonical binding site. Collectively, the docking simulations suggest that both lopinavir and ritonavir can be accommodated in the central cavity of hERG and directly binding to the channel via hydrogen bonds and hydrophobic interactions. The dockings also revealed the possibility that a phenyl group from any of the two drugs might enter a pocket under the selectivity filter and bind to F557.

### Remdesivir

**Figure 4:**
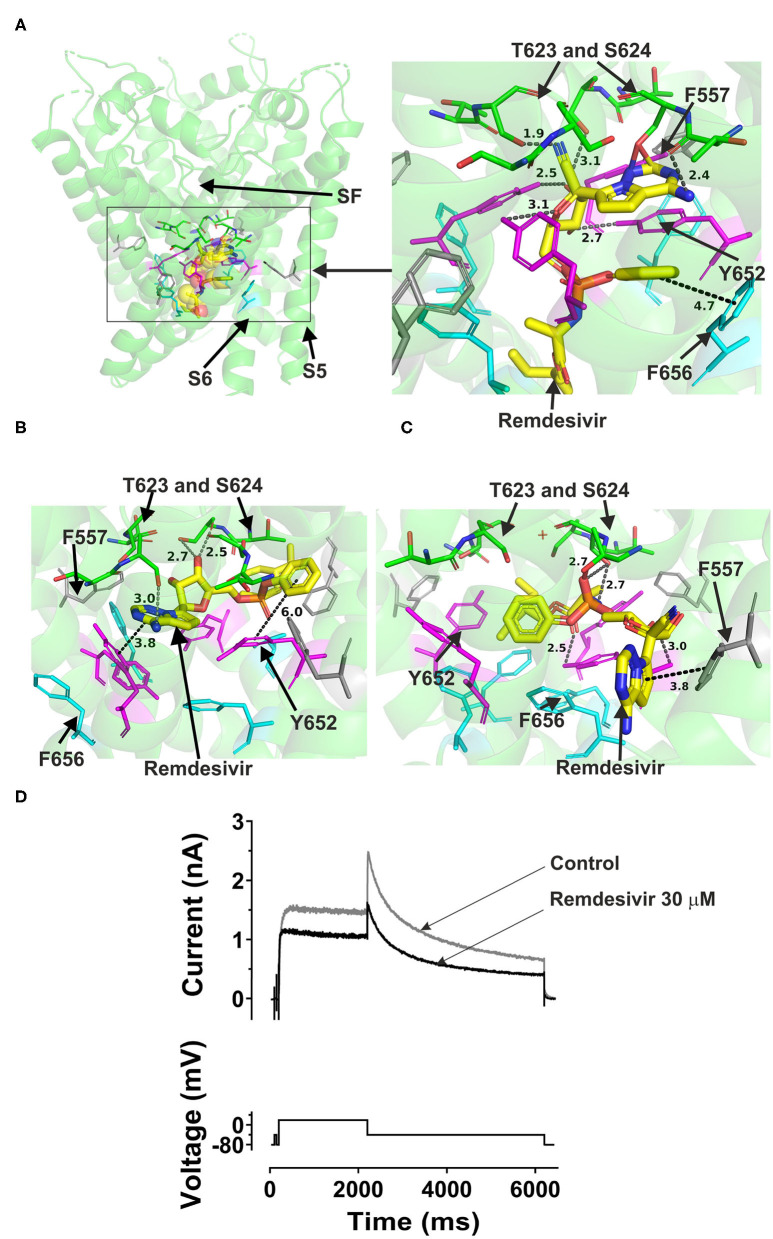
Location of the assigned pore binding site of remdesivir in the hERG cryo-EM structure and the closely related Dickson et al. model and the house MD model with the drug docked in the configuration shown in the methods section. The complete structure in **(A)** shows the location of segment 6 (S6) lining the pore where key residues such as Y652 and F656 are located and the outer S5 segment where F557, a major residue of the side pocket, is located. The selectivity SF was also annotated where T623 and S624 sit at the base of the pore helix attached to it. Remdesivir is represented as a space-filling *yellow* surface. Major amino acid constituents of the binding sites were shown as sticks. Binding residues and atoms of drug molecule colored as for Figure 1. **(A,B)** show low-energy-score pose for remdesivir docked into the hERG pore with docking biased to promote occupation of canonical binding site. Annotations (dotted lines) define potential interactions between drug and amino acid side chains, distances in A between the drug molecule and key residues were written adjacent to each dotted line. **(C)** shows the low-energy-score pose for remdesivir docked into the hERG pore with docking biased to promote occupation of the side pocket. **(A)** Shows the remdesivir docking in the hERG cryo-EM structure. **(B)** shows the remdesivir docking in the Dickson et al. model based on hERG cryo-EM structure. This run in the Dickson et al. model is particularly important since rotamers of at least one of Phe-656 side chains was selected to orient the side chain Cα-Cβ bond toward the pore. **(C)** shows the remdesivir docking in the in house MD hERG model where the drug was docked in the region of the side pocket. **(D)** I_hERG_ during superfusion with control (Tyrode's) solution and during application of 30 μM of remdesivir. I_hERG_ was elicited by a voltage protocol shown as lower traces, comprised of a 2 s depolarizing pulse to +20 mV, followed by repolarization to −40 mV. Thirty micomolar remdesivir inhibited I_hERG_ tails, producing a fractional block 0.38 ± 0.02, (i.e., a mean tail current amplitude reduction of 38%; *n* = 6).

Remdesivir could fit into both the canonical site in the central cavity and the side binding pocket in open channel hERG models (Figure 4). Docking the drug into the hERG cavity revealed potential binding of the drug to the channel protein via several hydrogen bonds and some hydrophobic interactions. The three docking runs in the channel open conformation (Cryo-EM structure, Dickson et al. and the in-house models) showed the drug can reside in the central cavity and mainly interact with Y652 in S6, L622, and S624 residues near the selectivity filter. The details of docking remdesivir in the cavity of either the open hERG represented by Dickson et al. model or the cryo EM structure are largely similar (details are in Supplementary Figure 5). However, distinct from the EM structure, docking to the Dickson et al. model showed the possibility of F656 in S6 interacting with remdesivir. More importantly, docking to the Dickson model showed the potential that part of the remdesivir molecule can advance toward the side pocket and interact with F557 (S5) and M651 (S6) and L622 from the pore helix. These residues are key amino acids in the side pocket (details are shown in Supplementary Figure 5.

Remdesivir was also docked to the in-house made open hERG model. When docked to the central cavity binding site, the molecule -as the above- described could be fitted in the cavity.

Interestingly, remdesivir was successfully docked in the side pocket in the in-house open model of hERG. The major aromatic parts of the structure which in previously described runs were residing in the cavity could access the binding pocket in this docking setting and able to interact with F557, L622, and Y652 (further details are in Supplementary Figure 6). This pose showed the possibility for remdesivir to be accommodated in and make interactions with the side pocket binding site while the other above three dockings to the open pore structure and models showed the potential interactions of remdesivir with key binding determinant in the central cavity. Like atazanavir, lopinavir, and ritonavir, remdesivir could not be accommodated in the closed hERG channel. In the period following initial submission/review of this report an independent study was published in which an acute inhibitory effect of remdesivir on hERG channels was reported to be absent (51). Therefore, a limited experimental series was conducted here to evaluate the effect of acute application of remdesivir on I_hERG_. The response to remdesivir was measured using the protocol shown in Figure 4D. This was comprised of a 2 s depolarization from−80 to +20 mV, followed by repolarization to −40 mV, at which the resurgent tail current that is typical of hERG was observed (52). I_hERG_ tail magnitude was measured as described previously (48–50, 52). Exemplar traces are shown in Figure 4D. 30 μM remdesivir inhibited hERG current by 38 ± 2% (*n* = 6).

### Favipiravir

**Figure 5:**
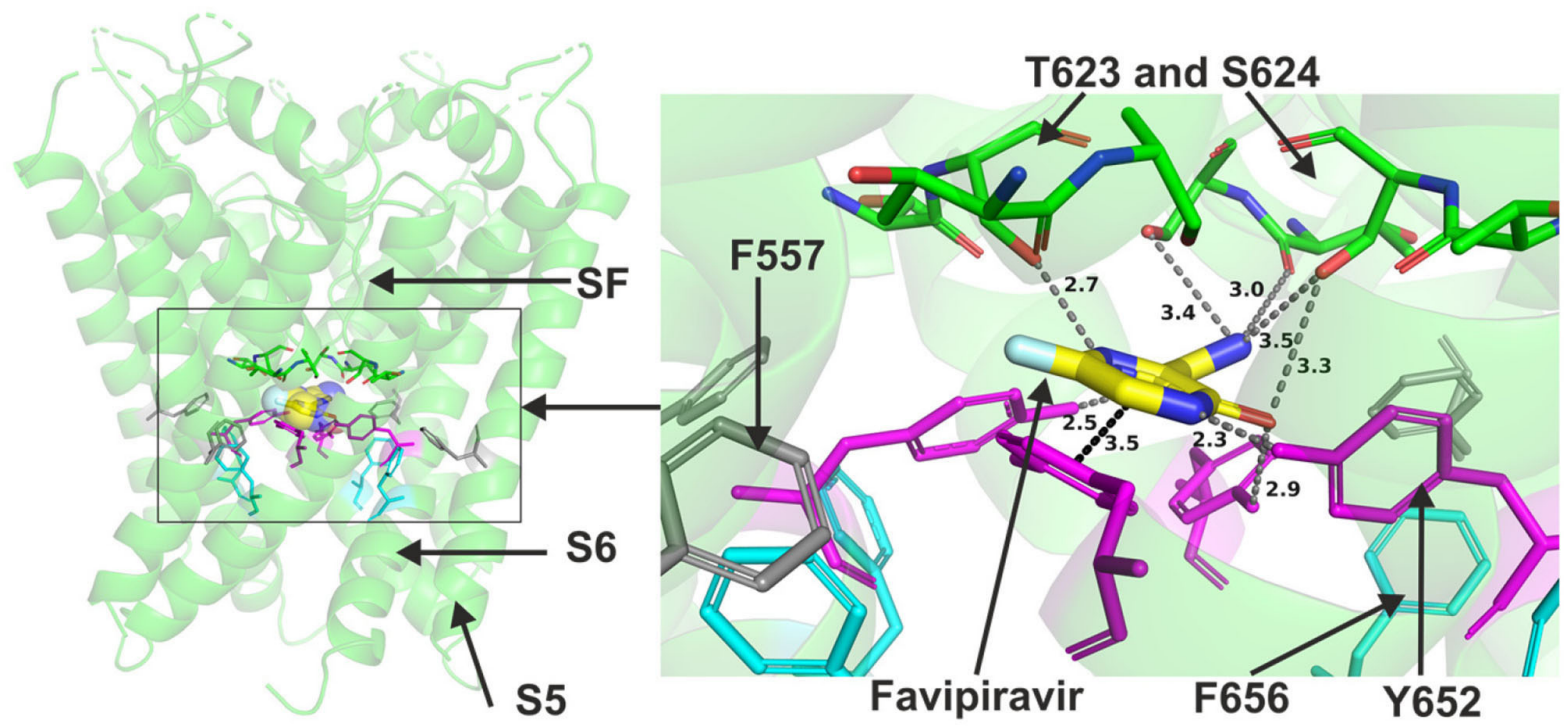
Location of the assigned binding site of favipiravir in the hERG cryo-EM structure. The complete structure shows the location of segment 6 (S6) lining the pore where key residues such as Y652 and F656 are located and the outer S5 segment where F557, a major residue of the side pocket, is located. The selectivity SF was also annotated where T623 and S624 sit at the base of the pore helix attached to it. Favipiravir is represented as a space-filling surface. Major amino acid constituents of the binding sites were shown as sticks in the box. Binding residues and atoms of drug molecule colored as for Figure 1. The figure shows a low-energy-score pose for favipiravir docked into the hERG pore with docking biased to promote occupation of canonical binding site. Annotations (dotted lines) define potential interactions between drug and amino acid side chains, distances in A between the drug molecule and key residues were written adjacent to each dotted line.

Favipiravir is a very small molecule (MW of 157 g/mol) compared to the other antivirals studied. Due to its small size, favipiravir was readily accommodated within the central cavity of open pore structure and models of hERG and the closed model of the channel (Figure 5). It could also fit to the side pocket of the in-house open pore model of hERG. However, favipiravir could only make relatively few binding contacts with the channel in all these dockings. The molecule also appeared relatively distant from key residues (Supplementary Figure 6). The residues involved in different poses include T623, S624, and Y652. The binding was slightly improved when docked to the side pocket which involved interaction with F557 (further details are in Supplementary Figure 7). Favipiravir also interacted weakly with the channel when docked in the closed model of hERG with the potential to interact with Y652, T623 and S624 residues (see also Supplementary Figure 7).

## Discussion

### Implications of the Findings of This Study

The results of this *in silico* study demonstrate that despite their comparatively large size, atazanavir, lopinavir, ritonavir, and remdesivir can interact with the canonical binding site on the hERG potassium channel. At present there are no *in vitro* mutagenesis data available for lopinavir, ritonavir, and remdesivir to verify that these drugs interact predominantly or solely with the pore binding site on hERG. However, the fact that our simulation data for atazanavir are consistent with experimental data that implicate the aromatic Y652 and F656 residues in hERG channel current (I_hERG_) blockade (39) provides confidence in the approach adopted here. Furthermore, to ensure the docking performance was consistent across software platforms, we also ran the docking procedure in the MOE suite (with a similar setting to Gold) where we found the docking energy and poses of the top poses were correlated with those in Gold (results not shown).

Our *in silico* data enable predictions to be made that can be addressed in future experimental studies. First, the inability of atazanavir, lopinavir, ritonavir, and remdesivir to interact with pore binding determinants in the closed channel state is consistent with a requirement for gating to occur for these agents to be able to interact with aromatic binding residues. Atazanavir has been reported not to alter voltage dependent activation or inactivation of wild-type (WT) hERG current (I_hERG_), but protocols to interrogate a requirement for channel opening were not applied (39). Similarly, detailed interrogation of the kinetics of lopinavir/ritonavir inhibition was not conducted (30). The results of our docking simulations suggest that it is likely that, with the potential exception of favipiravir, the drugs studied here can only access key binding determinants on channel gating; this should manifest in a measurable time-dependence of inhibition on channel opening.

The reported hERG current IC_50_ for atazanavir inhibition of I_hERG_ is 5.7 μM (39), whilst those for lopinavir and ritonavir are similar, being, respectively, 8.6 and 6.2 μM (30). In documents considered by the European Medicines Agency (EMA) early during the Covid-19 pandemic, for the compassionate licensing of remdesivir, its hERG IC_50_ is given as 28.9 μM, which is 26-fold the estimated free drug concentration (C_max_) of 1.1 μM at the proposed maximal clinical dose (53). However, after submission of this study, an independent report was published claiming that remdesivir does not produce an acute inhibition of I_hERG_ at 10 or 50 μM (51). In the same study, chronic application of remdesivir led to increased hERG expression and I_hERG_ amplitude, consistent with a potential for the drug to promote hERG channel trafficking (51). The ability of drugs to rescue misprocessed mutant hERG channels has previously been linked to hydrophobic interactions within the pore-cavity (54); thus, trafficking promotion by remdesivir without an ability to produce acute block would be highly notable. In our experiments, we observed 38% inhibition of I_hERG_ by 30 μM remdesivir, which is in fair agreement with the inhibitory potency in documents submitted to the EMA (53) and is inconsistent with a lack of acute I_hERG_ inhibition reported in (51). I_hERG_ inhibitory potencies of drugs can vary significantly depending on experimental temperature and stimulus waveform [e.g., (55, 56)]. Our measurements were made at 37°C, whilst those in (51) were made at room temperature, although whether or not this may account for the differences in respect of remdesivir is unclear. On the basis of comparison of I_hERG_ IC_50_ values and therapeutic C_max_ values of a broad range of drugs in relation to TdP risk, Redfern et al. proposed in 2003 a 30-fold safety margin for drugs undergoing clinical evaluation (57). A recent re-evaluation of the hERG safety margin for QT_c_ prolongation suggested an optimal margin of 50-fold (58). The safety margin for remdesivir may not exceed this value.

The single Ebola patient who experienced mild QT interval prolongation on favipiravir received multiple other drug treatments and experienced cardiac effusion (34); the factors that may have sensitized this patient to QT prolongation following favipiravir administration are unclear. In adult healthy volunteers, subjects oral dosing with 1,200 or 2,400 mg of favipiravir did not affect QT or QT_c_ intervals (33). There are currently no peer reviewed, published data on I_hERG_ inhibition by favipiravir. However, publicly available information at the Japanese Pharmaceuticals and Medical Devices Agency (PDMA) suggests no inhibitory effects of favipiravir on I_hERG_ at 40 or 200 μM and only an ~8% reduction at 1,000 μM (which concentration was described as ~3 times the human C_max_) (59). Although no experimental details are available for this information, it is suggestive of a low propensity of favipiravir to produce a pharmacological block of hERG channels, which is borne out by the docking simulation results in the present study. Drug size has previously been observed to be a significant determinant of inhibitory potency when comparing drugs of different sizes that share structural similarity. Thus, in a direct comparison, the I_hERG_ IC_50_ value of the antianginal and antiarrhythmic agent ranolazine was ~16 fold lower than that of structurally similar, but smaller lidocaine (48); the difference was attributable to the fact that ranolazine was able to form a greater range of interactions with hERG pore residue side chains than was lidocaine (48). Whilst it is important that the effects of favipiravir on I_hERG_ are established under a known, standardized set of conditions and compared with other candidate antivirals, it seems likely that the small size of favipiravir may be advantageous in conferring comparatively low hERG liability.

### Relevance to Interrogation of Interactions of Drug Molecules With the hERG Pore Structure Determined With Cryo-EM

The publication of the cryo-EM structure revealed two unexpected structural features of the hERG channel: first, the central pore cavity of the channel was found to have a smaller volume compared to that assumed from homology modeling; second, four deep hydrophobic pockets surrounding the cavity were identified that could provide drug interaction sites (40, 41, 60). However, the cryo-EM structure represents a single, fixed hERG conformation and, at least for some drug molecules it has been difficult to recapitulate aspects of experimental mutagenesis data using the original cryo-EM structure (47, 61). For example, high potency I_hERG_ inhibition by the minimally structured hERG inhibitor “Cavalli-2” showed a strong sensitivity to mutation of F566, but in the cryo-EM structure the aromatic side chain of this residue was oriented away from the cavity (47). Reconciliation of docking with mutagenesis results required a small clockwise rotation of the S6 helix to optimize F656 residue orientations compatible with high affinity inhibition block (47). Here we employed both the original and modified cryo-EM structures. The use of different models produced a common outcome in that they all supported the ability of the antivirals studied to interact with the pore binding site; however, some drug- and model-specific observations were made. For example, of the larger antiviral molecules studied only remdesivir showed a propensity to interact with the lateral binding pockets surrounding the central cavity and there was a marked difference between interactions with residues in this region observed using the MD based Dickson model (42) and the original cryo-EM structure (40). For ritonavir, the use of the Dickson model allowed the drug to be in close proximity to F557 [a residue implicated in binding of a number of drugs (47, 62–64)]. The future experimental investigation of pore cavity and lateral binding pocket residue mutants should be able to identify which of the different binding modes predicted here most accurately describes drug-channel interactions and whether or not any particular channel structure is used for the docking here outperforms the others in matching experimental observations. Moreover, the inability of atazanavir, lopinavir, and ritonavir to reside in the lateral pockets of the cryo-EM structure, should make these drugs valuable for comprehensive (alanine-scanning) mapping of binding to the channel pore, with a general lack of responsiveness to mutation of residues predicted to line the lateral pockets.

### Limitations and Conclusions

This study was conducted almost entirely *in silico* and was designed to investigate potential interactions between the selected drugs and hERG only with the canonical drug binding site and lateral pockets that can inform future experimental studies. Given the comparatively large size of most of the drugs studied, we cannot preclude the potential for (additional) interactions outside the channel pore, as may occur for macrolide antibiotics (65).

hERG liability is a very important consideration but not the only one in the evaluation of pro-arrhythmic risk with clinically used drugs. Potential drug effects on other channels that might mitigate the effects of hERG block need to be considered for an overall evaluation of cardiac risk (36, 37, 66). Whilst it is important to acknowledge these limitations, the strengths of the present study are that it: (i) highlights the potential for all the drugs studied here to interact with hERG; (ii) provides specific observations that can form the basis for experimental hypothesis formation and testing; and consequently (iii) provides a valuable basis from which future experimental investigation of both hERG inhibition and overall cardiac arrhythmia liability can be tested. This may be particularly important for remdesivir and favipiravir, given their potential as COVID-19 treatments. Indeed, the present study usefully complements a recent independent investigation that has used a combination of predictive indices for drug-induced LQTS (though not structural modeling as conducted here) to evaluate risks with potential COVID-19 treatments, on the basis of which it has recommended close monitoring of QT/QT_c_ intervals in patients receiving both drugs (67). On the basis of our observations, we suggest that a direct *in vitro* experimental comparison would be informative of I_hERG_ inhibitory potency between remdesivir, atazanavir, lopinavir, and ritonavir and favipiravir under a standardized set of conditions; this would aid further evaluation of likely I_hERG_ safety margin. Those data could usefully be combined with further acute and chronic channel assays employing additional key ventricular ion channels and action potential repolarization measurements to arrive at an integrated preclinical risk evaluation. Finally, it should be noted that whilst the motivation for this study arose from ongoing efforts toward the repurposing of the drugs studied here for COVID-19, any implications for cardiac safety also have wider relevance for the use of these agents in the treatment of other infectious conditions.

## Data Availability Statement

The original contributions presented in the study are included in the article/Supplementary Material, further inquiries can be directed to the corresponding author/s.

## Author Contributions

JH and EA-M conceived and designed the study and drafted the manuscript. EA-M and MS conducted and analyzed the docking simulations. EA-M conducted patch clamp recording. All authors revised the manuscript.

## Conflict of Interest

The authors declare that the research was conducted in the absence of any commercial or financial relationships that could be construed as a potential conflict of interest.
