# Supplementary material for: In silico Exploration of Interactions Between Potential COVID-19 Antiviral Treatments and the Pore of the hERG Potassium Channel—A Drug Antitarget
Source: Front Cardiovasc Med. 2021 May 4;8:645172. doi: 10.3389/fcvm.2021.645172 (PMC8129016; doi:10.3389/fcvm.2021.645172)
Supplement: Supplementary file 1 [file Data_Sheet_1.PDF]

Online supplement for

In silico exploration of interactions between potential COVID-19 antiviral treatments and the pore of the hERG potassium channel– a drug antitarget

Ehab Al-Moubarak[1+], Mohsen Sharifi[2+], and Jules C Hancox[1*]

[1]School of Physiology, Pharmacology and Neuroscience, Biomedical Sciences Building, University Walk, Bristol, BS8 1TD.

[2]Independent Scientist. 1844 Century way, Ste 3, Indianapolis, Indiana 46260, USA

Corresponding author at: jules.hancox@bristol.ac.uk

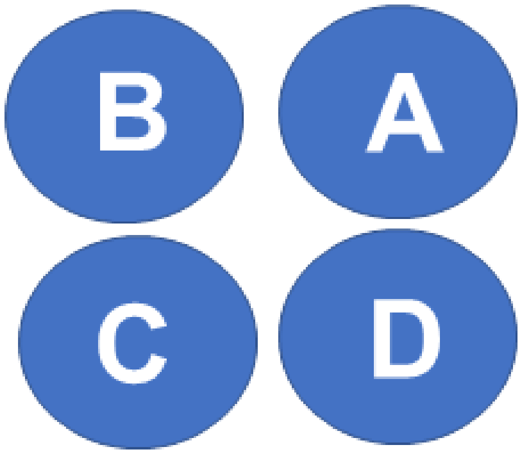

Figure S1 hERG subunit labelling

Schematic diagram showing top-down view of hERG tetramer. Functional hERG channels are comprised of four identical subunits. Drug-channel interactions between molecules in the pore and residues comprising the binding site can involve multiple interactions with the same subunit or interactions with residues from different subunits of the tetramer. In the main text and supplementary figures interactions with residues on different subunits are denoted by the use of subunit labelling according to this schematic, using the notation "subunit: residue" (e.g. C:F557).

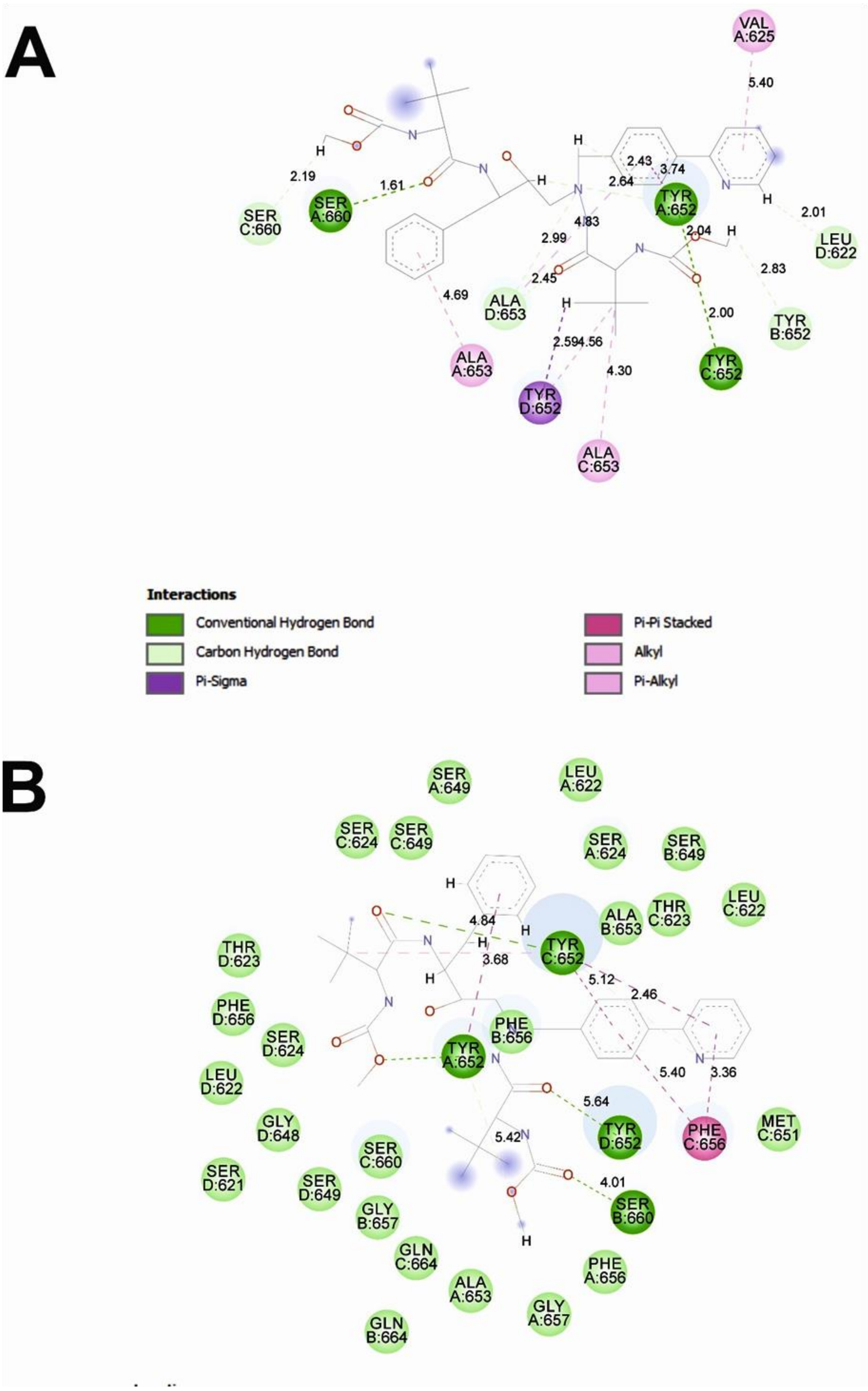
A
VAL A:625
5.40
2.19
1.61
SER C:660
SER A:660
2.43
3.74
2.64
TYR A:652
2.01
LEU D:622
4.83
2.99
2.04
2.45
2.83
4.69
ALA D:653
TYR B:652
2.00
2.594.56
ALA A:653
4.30
TYR C:652
TYR D:652
ALA C:653
Interactions
Conventional Hydrogen Bond
Carbon Hydrogen Bond
Pi-Sigma
Pi-Pi Stacked
Alkyl
Pi-Alkyl
B
SER A:649
LEU A:622
SER C:624
SER C:649
SER A:624
SER B:649
LEU C:622
4.84
ALA B:653
THR C:623
THR D:623
TYR C:652
3.68
5.12
2.46
PHE D:656
PHE B:656
SER D:624
TYR A:652
5.40
3.36
LEU D:622
5.64
GLY D:648
5.42
TYR D:652
PHE C:656
MET C:651
SER C:660
4.01
SER D:621
SER D:649
SER B:660
GLY B:657
GLN C:664
PHE A:656
ALA A:653
GLY A:657
GLN B:664

Figure S2: Atazanavir dockings in the open channel hERG models Atazanavir was predicted to interact with hERG in the open channel conformation when docking was biased towards the canonical binding region. The Figure shows the likely interactions and predicted binding amino acids. The interaction types are denoted by colours as shown in the legend within the Figure. Distances between interacting moieties are shown next to the dotted lines connecting interacting atoms in angstroms. A: The top pose in the docking run in Cryo-EM hERG structure B: The top pose in the docking run in Dickson et al model.

Docking atazanavir in *various* open hERG models showed the possibility that the drug may contact the channel protein by various hydrogen bonds and hydrophobic interactions. In both the Cryo EM hERG structure (Figure S2A) and the Dickson et al model (Figure S2B), atazanavir were able to form hydrogen bonds with Y652 and S660 mainly with methoxycarbonyl, butanoyl, and carbamate groups. These were as follows: Cryo-EM: A: S660 with the methoxycarbonyl group, A:Y652 and C: Y652 with the carbamate group, in Dickson et al model: A:Y652 with the methoxycarbonyl group, C: Y652 with the butanoyl group. However, in the Cryo-EM structure, the pyridine group could also form a hydrogen bond with D:L622. In both hERG structure presentations, atazanavir made abundant hydrophobic interactions owing to its three aromatic rings. Residues involved in these hydrophobic interactions involve Y652, and F656. In the cryo EM structure: A: Y652 formed a pi-pi stacked interaction with the phenyl group attached to pyridine D: Y652 formed Pi stigma with a methyl group attached to the oxobutan group. A: V625 formed alkyl/ pi alkyl with the pyridine group. In the Dickson et al model: C: Y652 and C: F656 formed pi-pi stacked interactions with the pyridine and phenyl group attached to it. A:652 formed pi-pi stacked interaction with the phenyl group attached to the butyl group within atazanavir molecule**.** Atazanavir was also docked in a side pocket under the selectivity filter in the open pore state of the channel. However, due to its size, the drug could not fit in the binding pocket.

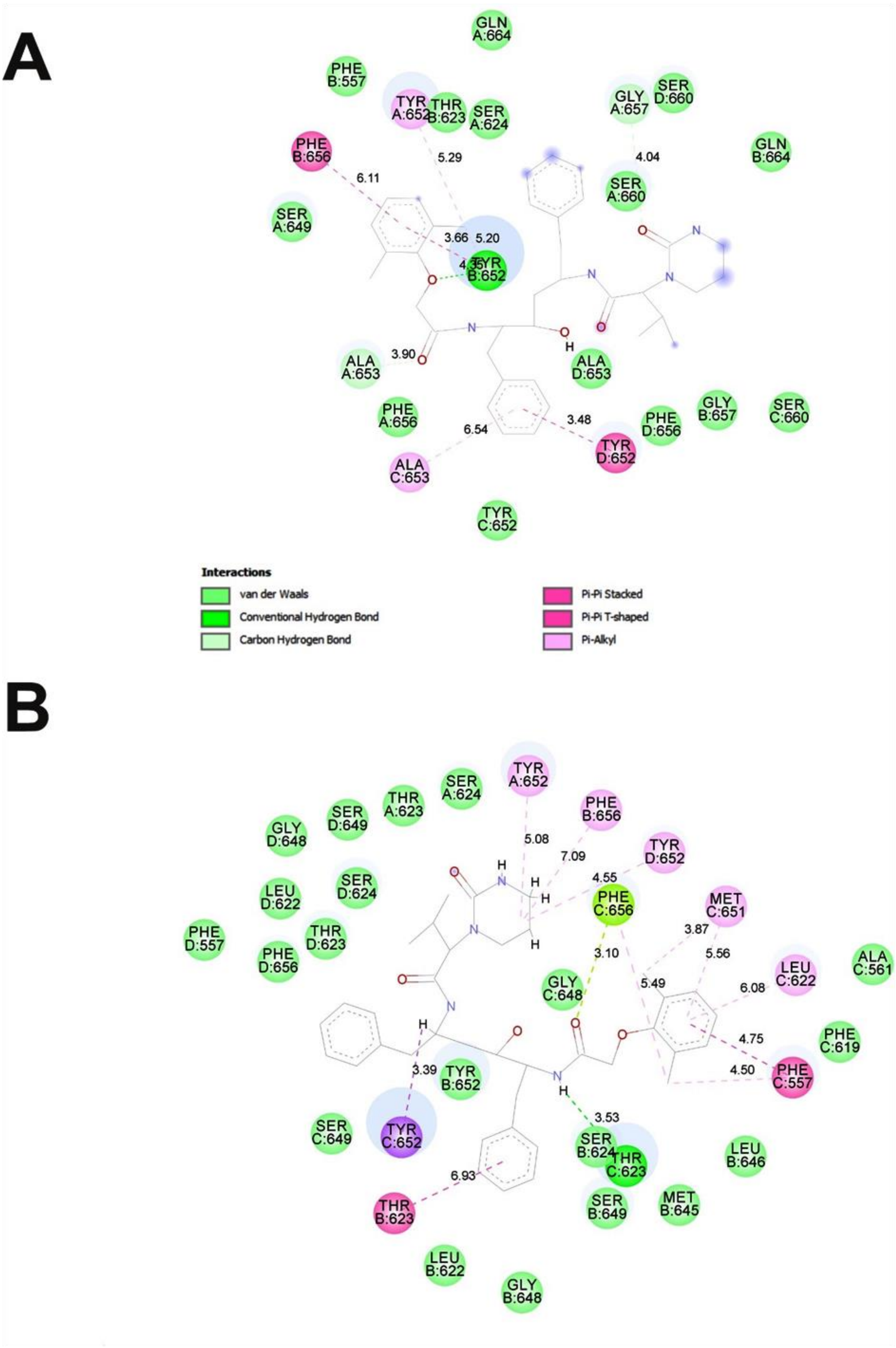
A
GLN A:664
PHE B:557
TYR A:652
THR B:623
SER A:624
GLY A:657
SER D:660
GLN B:664
PHE B:656
SER A:660
SER A:649
TYR B:652
ALA A:653
ALA D:653
PHE A:656
GLY B:657
SER C:660
PHE D:656
TYR D:652
ALA C:653
TYR C:652
6.11
5.29
4.04
3.66
5.20
3.90
6.54
3.48
Interactions
van der Waals
Conventional Hydrogen Bond
Carbon Hydrogen Bond
Pi-Pi Stacked
Pi-Pi T-shaped
Pi-Alkyl
B
TYR A:652
SER A:624
THR A:623
SER D:649
GLY D:648
PHE B:656
TYR D:652
LEU D:622
SER D:624
PHE C:656
MET C:651
PHE D:557
THR D:623
PHE D:656
GLY C:648
LEU C:622
ALA C:561
PHE C:619
PHE C:557
TYR B:652
TYR C:652
SER C:649
SER B:624
THR C:623
LEU B:646
THR B:623
SER B:649
MET B:645
LEU B:622
GLY B:648
5.08
7.09
4.55
3.87
3.10
5.56
5.49
6.08
4.75
4.50
3.39
3.53
6.93

Figure S3: Lopinavir dockings in the open channel hERG models Lopinavir was predicted to interact with hERG in the open channel conformation when docking was biased towards the canonical binding region. The figure shows the likely interactions and predicted binding amino acids. The interaction types are denoted by colours as shown in the legend within the figure. Distances between interacting moieties are shown next to the dotted lines connecting interacting atoms in Angstroms. A: The top pose in the docking run in Cryo EM hERG structure B: The top pose in the docking run in Dickson et al model.

Lopinavir fitted well in the central cavity of the hERG open channel. B: F656, D: Y652 and B:Y652 were able to make pi-pi interactions with phenyl groups within the drug in the low energy poses in the cryo EM structure (Figure S3A) and similarly in the open pore in-house model. Docking lopinavir into the open channel model by Dickson et al (Figure S3B) resulted in a phenyl group extending towards the side pocket and interact with C:F557 by pi-pi stacking interaction. In this pose, B:T623 also interacted with another phenyl group by an amide-pi stacking interaction. Lopinavir, in the same pose, still able interacted with key residues in the canonical binding site: a methyl group made a pi-sigma interaction with Y652 while F656 interacted with an oxygen atom in the amide group via pi-lone pair interaction.

Docking of lopinavir to open hERG models revealed the possibility that the drug can also form hydrogen bonds with the channel, mainly with S624, F656 and Y652. Lopinavir could bind to B: S624 in hERG via a hydrogen bond with an amide group in the drug in the least energy docking pose following docking in the Cryo EM structure variant by Dickson et al. Also, C: S624, appeared to be able to contact the drug via van der Waals interactions with hydrogen atoms in the diazinan group of lopinavir as predicted by docking in the in-house open pore hERG model. Both docking in Dickson and this in-house model revealed that the aromatic F656 residue can form a hydrogen bond or Pi-Lone pair interactions with nearby oxygen atoms within the drug. Docking to the original cryo EM open hERG structure also suggested hydrogen interactions with the drug can be formed. B:Y652 made a hydrogen bond with the phenoxy group of the drug. In the same pose, A: G657 also appeared to contact the drug molecule via van der Waals interaction with the oxygen atom in the diazinan group.

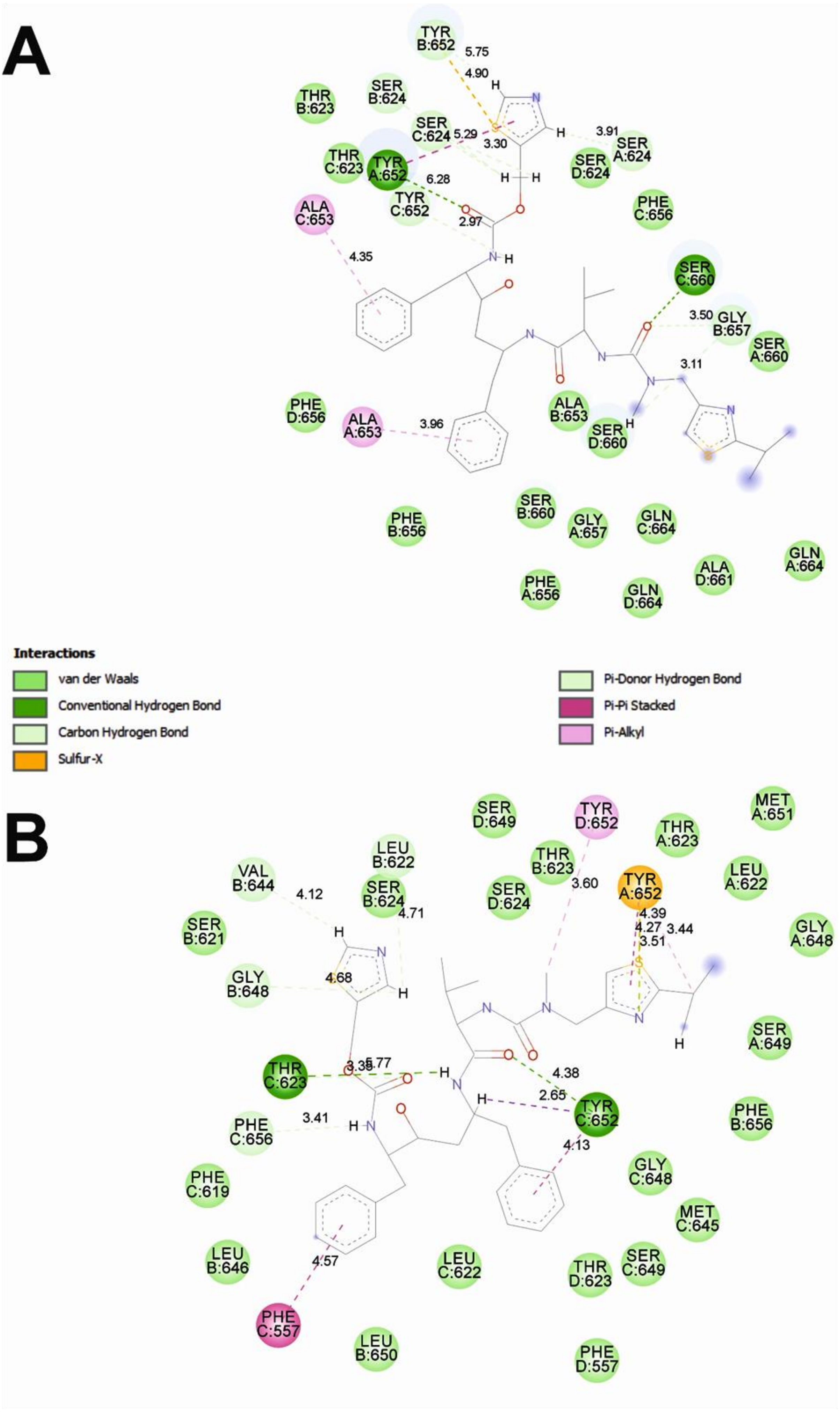
A
TYR B:652
5.75
4.90
THR B:623
SER B:624
SER C:624
5.29
3.30
3.91
SER A:624
SER D:624
THR C:623
TYR A:652
6.28
TYR C:652
ALA C:653
2.97
PHE C:656
4.35
SER C:660
3.50
GLY B:657
SER A:660
3.11
PHE D:656
ALA A:653
3.96
ALA B:653
SER D:660
SER B:660
PHE B:656
GLY A:657
GLN C:664
GLN A:664
ALA D:661
PHE A:656
GLN D:664
Interactions
van der Waals
Conventional Hydrogen Bond
Carbon Hydrogen Bond
Sulfur-X
Pi-Donor Hydrogen Bond
Pi-Pi Stacked
Pi-Alkyl
B
SER D:649
TYR D:652
MET A:651
THR A:623
LEU B:622
THR B:623
VAL B:644
4.12
SER B:624
4.71
SER D:624
3.60
TYR A:652
LEU A:622
4.39
4.27
3.44
3.51
SER B:621
GLY A:648
GLY B:648
4.68
SER A:649
THR C:623
4.38
2.65
TYR C:652
PHE C:656
3.41
PHE B:656
4.13
GLY C:648
PHE C:619
MET C:645
LEU B:646
4.57
LEU C:622
THR D:623
SER C:649
PHE C:557
LEU B:650
PHE D:557

Figure S4: Ritonavir dockings in the open hERG models Ritonavir was predicted to interact with hERG in the open channel conformation when docking was biased towards the canonical binding region. The figure shows the likely interactions and predicted binding amino acids. The interaction types are denoted by colours as shown in the legend within the figure. Distances between interacting moieties are shown next to the dotted lines connecting interacting atoms in Angstroms A: The top pose in the docking run in Cryo EM hERG structure B: The top pose in the docking run in Dickson et al model.

Ritonavir could be docked in the canonical binding site of hERG in both the Cryo-EM structure (Figure S4A) and the Dickson et al model (Figure S4B), both representing the open pore state of the channel.

Docking ritonavir to the cryo-EM structure predicted interactions with several residues including Y652, S660 and S624 in the central cavity of the channel. The A:Y652 was able to interact with the drug via hydrogen bond with the carbamate group in the drug and also via pi-pi stacked hydrophobic interaction with a nearby thiazole group. In the same pose, the pore helix residue S624 was forming hydrogen bonds with the same thiazole group. C:S660 and B:G657 were also found to form a hydrogen bond with an amide group.

Docking the drug in the Dickson et al model revealed a slightly different pose in which a phenyl group from the drug was able to advance near the peripheral residue C:F557 forming a pi-pi stacked hydrophobic interaction. In this pose, Y652 residue from two different subunits (C and A) were also able to form pi-pi stacked interactions with a phenyl group and with a thiazole group. T623, S624 and F656 could interact with the drug via hydrogen bonds. Y652 may interact with the sulphur atom in a thiazole group within the ritonavir molecule as predicted in both binding models.

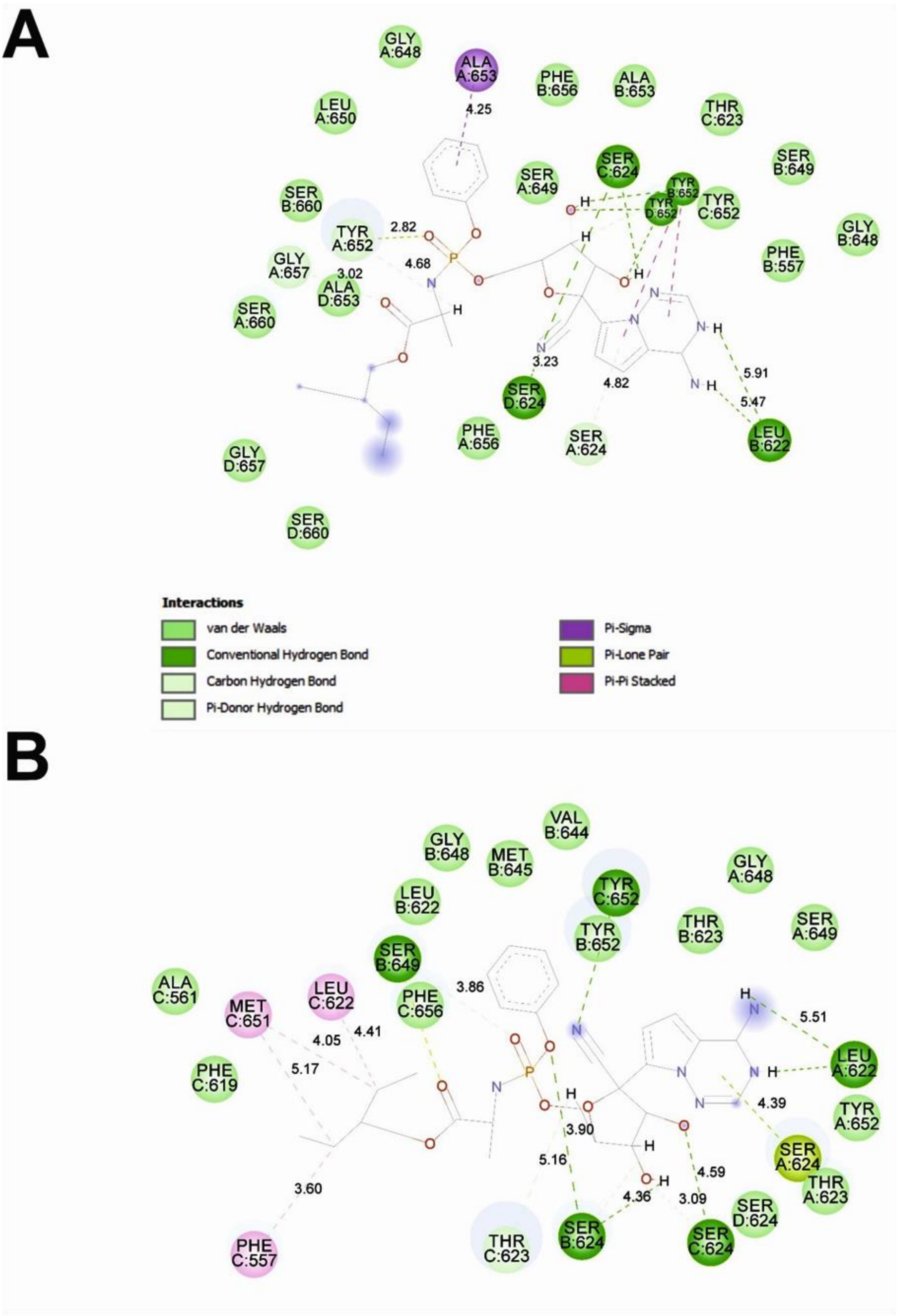

Figure S5: Remdesivir dockings in the open hERG models Remdesivir was predicted to interact with hERG in the open channel conformation when docking was biased towards the canonical binding

region. The figure shows the likely interactions and predicted binding amino acids. The interaction types are denoted by colours as shown in the legend within the figure. Distances between interacting moieties are shown next to the dotted lines connecting interacting atoms in Angstroms. A: The top pose in the docking run in Cryo EM hERG structure B: The top pose in the docking run in Dickson et al model.

Remdesivir was successfully docked in the central cavity of the EM structure (Figure S5A), and in the Dickson et al model (Figure S5B). It can also fit into the side binding pocket in open channel hERG models as per docking to the inhouse MD model (Figure S6). Docking the drug into the hERG cavity revealed potential binding via several hydrogen bonds and some hydrophobic interactions. The binding models involved the central cavity showed that the structural region of remdesivir which involved the pyrrole, triazine and oxolan aromatic groups can reside and mainly interact with the central cavity residues Y652, L622 and S624.

When remdesivir was docked to the cryo-EM open hERG structure, interactions involved the pyrrole, triazine and oxolan groups from remdesivir with key residues from the pore helixes and inner helixes contributing from hERG. These residues included L622, S624 and Y652. In this lowest energy pose, B: L622 formed two hydrogen bonds with triazine and amino group attached to triazine. S624 from two subunits (D and C) formed hydrogen bonds with the cyano group and hydroxy group attached to the oxolan group. D: Y652 was also able to form hydrogen bonds with the two hydroxy groups attached to the oxolan group. In this pose, there was potential for B: Y652 to form pi-pi stacked hydrophobic interactions with the pyrrole and the triazine groups. Docking remdesivir in the cryo-EM structure also showed the possibility that A:Y652 residue may form a pi- lone interaction with the phosphoryl group within the drug's molecule.

Docking remdesivir in the cavity of the open hERG, Dickson et al model identified similar binding possibilities to those with the cryo EM structure concerning the structural region containing the aromatic pyrrole, triazine and oxolan groups. However, distinct from the EM structure, docking to the Dickson et al model showed the possibility of C: F656 interaction with the propanoate group via a hydrogen bond. Also, it showed the potential of this propanoate group and the methyl group attached to it to advance towards the binding pocket and interact with C:F557 and C:M651 via alkyl/ pi alkyl interaction, which was simultaneously involving contact with the C:L622 from the pore helix. Hydrogen bond contacts played a major role in the drug interaction with the channel. S624 residues from different chains (B and C) formed hydrogen bonds with hydroxy groups attached to the oxolan group as well as to the

phosphoryl group. A:S624 may also form a pi lone pair with the triazine group. Identical to docking to the cryo EM structure, A:L622 formed two hydrogen bonds with triazine and amino group attached to triazine. This binding model also involves a hydrogen bond between C:Y652 and the cyano group.

Remdesivir was also docked to the in house made open hERG model binding the drug to the central cavity. In this pose, C:Y652 formed pi-pi stacked hydrophobic interactions with the pyrrole and the triazine groups. B:S624 formed a hydrogen bond with the cyano group. While the B:F656 interacted with the further part of the structure near the propanoate group.

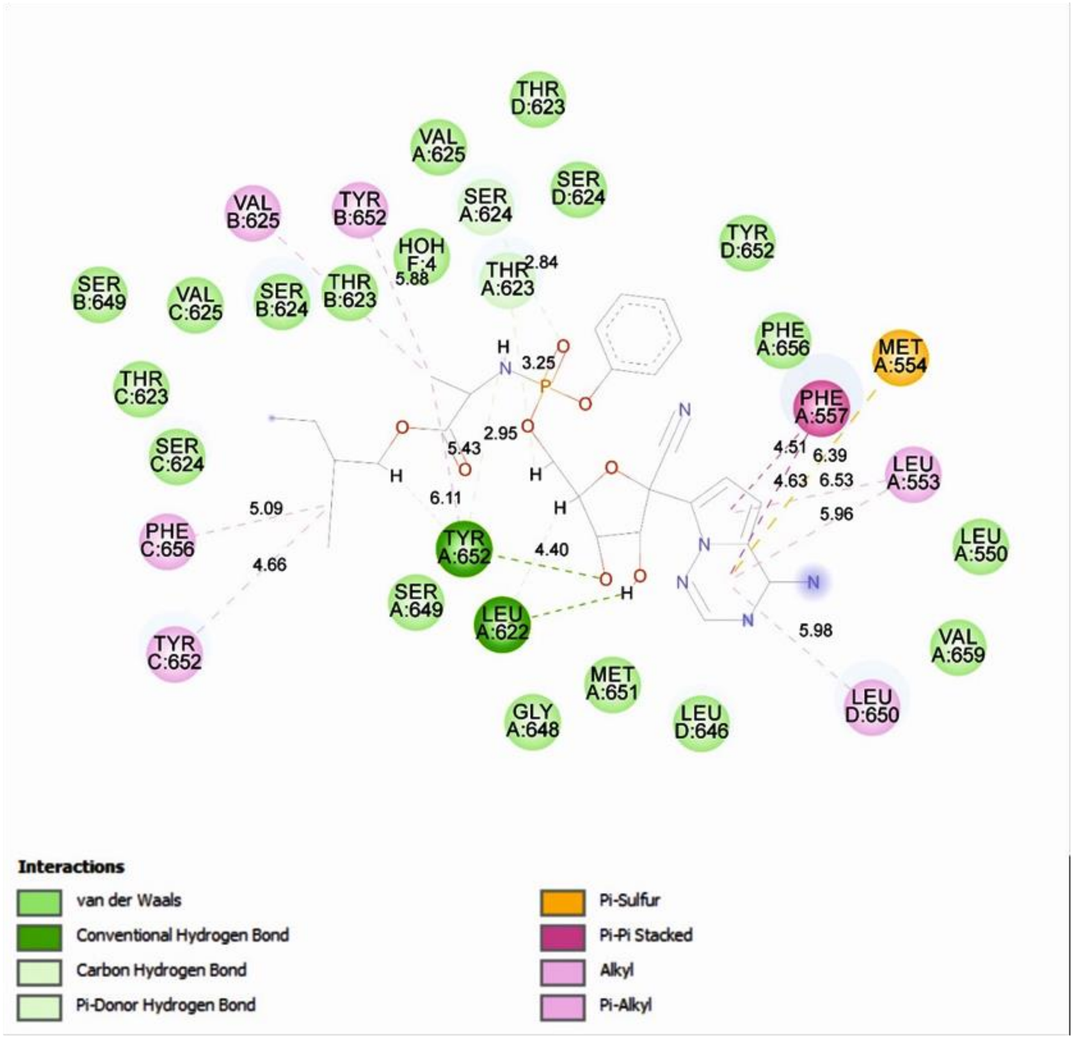

Figure S6: Remdesivir dockings in inhouse open hERG model Remdesivir was predicted to interact with hERG in the open channel conformation when docking was biased towards the side pocket. The figure shows the likely interactions and predicted binding amino acids. The interaction types are

denoted by colours as shown in the legend within the figure. Distances between interacting moieties are shown next to the dotted lines connecting interacting atoms in Angstroms.

Remdesivir was directly been docked in the binding pocket in the in-house open model of hERG (Figure F6). The drug was accommodated in the side pocket and can interact with F557, L622 and Y652. The lowest energy pose showed A:F557 interact with the pyrrole and the triazine groups via pi-pi stacked hydrophobic interactions. A:L553 and D: L650 also could contact the groups in this pose via alkyl/pi-alkyl interactions. A:L622 and A:Y652 was able to form hydrogen bonds with the hydroxy groups attached to the oxolan group. Peripheral parts of the remdesivir structure near and including the propanoate group could interact with B: V625, Y652 (B and C) and C: F656 via alkyl/pi-alkyl interactions. Docking remdesivir into the side pocket showed the possibility that the major aromatic bulk of its molecule can also reside in the side pocket and perfectly interact with the binding determinant in this structural region.

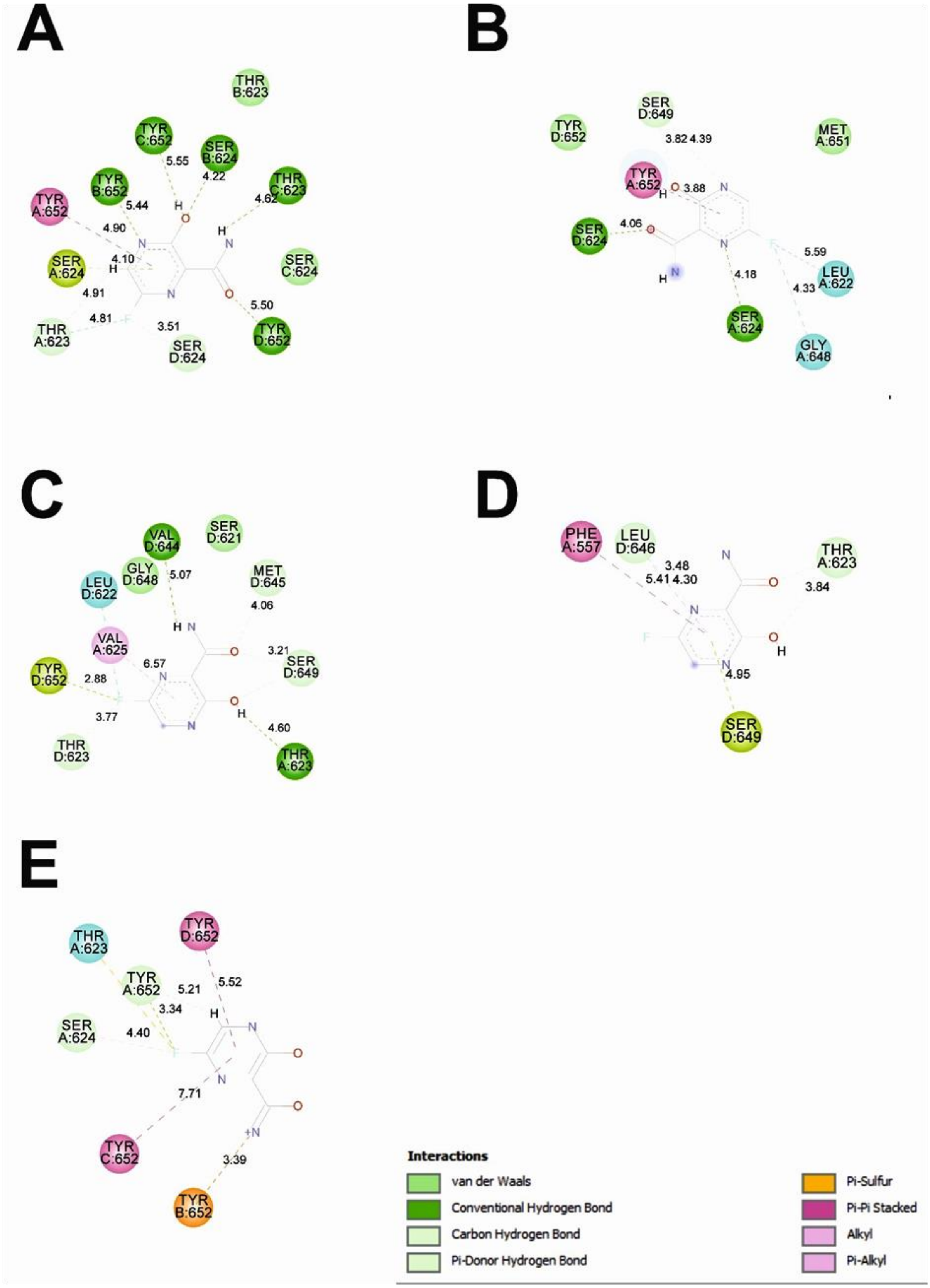
A
THR B:623
TYR C:652
SER B:624
THR C:623
TYR B:652
TYR A:652
SER A:624
SER C:624
THR A:623
SER D:624
TYR D:652
5.55
4.22
4.62
5.44
4.90
4.10
4.91
4.81
3.51
5.50
B
SER D:649
TYR D:652
MET A:651
TYR A:652
SER D:624
LEU A:622
SER A:624
GLY A:648
3.82 4.39
3.88
4.06
5.59
4.18
4.33
C
VAL D:644
SER D:621
GLY D:648
LEU D:622
MET D:645
VAL A:625
TYR D:652
SER D:649
THR D:623
THR A:623
5.07
4.06
6.57
3.21
2.88
3.77
4.60
D
PHE A:557
LEU D:646
THR A:623
SER D:649
3.48
5.41 4.30
3.84
4.95
E
THR A:623
TYR D:652
TYR A:652
SER A:624
TYR C:652
TYR B:652
5.21
5.52
3.34
4.40
7.71
3.39
Interactions
van der Waals
Conventional Hydrogen Bond
Carbon Hydrogen Bond
Pi-Donor Hydrogen Bond
Pi-Sulfur
Pi-Pi Stacked
Alkyl
Pi-Alkyl

Figure S7: Favipiravir molecular docking in hERG models. Favipiravir was predicted to weakly interact with hERG in different channel conformations. The figure shows the likely interactions and predicted binding amino acids. The interaction types are denoted by colours as shown in the legend within the figure. Distances between interacting moieties are shown next to the dotted lines connecting interacting atoms in Angstroms. The top poses are shown in the docking runs. A: In Cryo EM hERG structure when docking was biased towards the canonical binding region. B: In Dickson et al model when docking was biased towards the canonical binding region. C: In the inhouse MD open model when docking was biased towards the canonical binding region. D: In the inhouse MD open model when docking was biased towards the side pocket binding region. E: In the closed hERG model when docking was biased towards the canonical binding region

Favipiravir was relatively very small molecule mainly comprised of a pyrazine group to which are attached hydroxy, fluorine and a carboxamide groups, and made weak contacts with hERG and appeared relatively distant in all docking configurations and binding models.

Docking favipiravir to the cryo EM structure showed potential hydrogen bonds between the carboxamide group and C:T623; D:Y652 (Figure S7A). Also, B:S624 appeared in this pose forming a hydrogen bond with the hydroxy group in the drug. C:Y652 may also form another hydrogen bond with the hydroxy group in favipiravir. A:T623 formed a halogen contact with the fluorine. It is also possible that A:Y652 forms pi pi stacked hydrophobic interaction with the pyrazine group. While docking in the cryo EM resulted in the best pose for favipiravir, the drug was only able to contact some of the pore helix residues (T623 and S624) and the nearby aromatic residue Y652.

Docking favipiravir to the Dickson et al model (Figure S7B) largely resulted in a similar outcome to that obtained from the cryo EM structure binding model. S624 from two subunits (D and A) formed hydrogen bonds with the carboxamide and the pyrazine groups. A:L622 and A:G648 formed a halogen interaction with the fluorine. Also, similarly, the aromatic A:Y652 formed pi pi stacked interaction with the pyrazine group.

Docking the drug to the in house MD open model in the central cavity showed a further weak binding profile (Figure S7C). This is featured with hydrogen bonds between the A:T623 and the hydroxy group on one side and between the D:V644 and the carboxamide group but lack any hydrophobic interaction in this predicted pose. The binding slightly improved when docking in the side binding pocket of this model (Figure S7D). A:F557 formed pi pi stacked

interaction with the pyrazine group. D:S649 in segment 6 of hERG may form pi lone pair also with the same group.

Favipiravir could also be fitted in the channel when docked in the closed model of hERG but with potentially weak interactions (Figure S7E). Y652 (C and D) were involved in pi pi stacked interactions and there was a potential of interactions between the fluorine and the A:T623 and A: S624.
